# A mixed methods study of the awareness and management of familial hypercholesterolaemia in Irish general practice

**DOI:** 10.3389/fmed.2022.1016198

**Published:** 2022-10-12

**Authors:** Robyn Homeniuk, Joseph Gallagher, Claire Collins

**Affiliations:** ^1^Research Centre, Irish College of General Practitioners, Dublin, Ireland; ^2^Cardiovascular Clinical Lead, Irish College of General Practitioners, Dublin, Ireland

**Keywords:** primary care, general practice, familial hypercholesterolaemia, hyperlipidemia, record screening, LDL cholesterol

## Abstract

**Introduction:**

Familial Hypercholesterolemia (FH) is one of the most common genetic disorders, with an estimated global prevalence of 1:200-500, which leads to premature cardiovascular disease. Nevertheless, public and professional awareness of FH is often lacking, with an estimated 20,000 largely undiagnosed cases in Ireland.

**Purpose:**

The overall aim of the project was to test the feasibility of a model of care that would include electronic record screening, clinical assessment, and coding of possible FH patients across a network of general practices in Ireland. In addition, a secondary aim was to gauge the awareness and knowledge of FH across the network.

**Methods:**

This study took part in multiple phases, employing a mixed methods design. The study included a validated questionnaire, tailored online educational resources, a retrospective chart review of patients with a history of elevated LDL cholesterol (LDLc) and an active review with a selection of those patients. Results were analyzed using SPSS V27, where descriptive statistics and relevant correlation tests were employed.

**Results:**

Eighteen general practices agreed to take part in the study. In the initial survey, respondents rated their personal and practice familiarity with FH as slightly below average. Around one-third of respondents were not aware of FH guidelines. Of over 55,000 adult patient records searched, only 0.2% had a recorded FH diagnosis and 3.9% had ever had an LDLc above 4.9 mmol/l. Eight practices completed 198 chart reviews. Among these, 29.8% of patients had a family history recorded, and 22.2% had a family history of CVD recorded. Female patients had higher averages for highest and recent LDLc. Seventy patients underwent a clinical review—with 27% of these patients identified as “probable” or “definite FH.” There was a statistically significant (*p* = 0.002) relationship between FH status and whether the patient had other CVD risk factors.

**Conclusion:**

General practitioners in Ireland had similar levels of awareness of FH compared to findings from elsewhere. The activities discussed encouraged clinicians to consider FH when talking to their patients, especially those with elevated LDLc at an early age. Broader awareness of the condition could increase conversations about FH and benefit patient outcomes.

## Introduction

Familial Hypercholesterolemia (FH) is an inherited or genetic disorder that leads to premature atherosclerotic cardiovascular disease. FH now presents a major public health concern, as untreated FH significantly results in higher and earlier risk for cardiovascular disease (1, 2). However, there is a documented lack of awareness among both the public and health professionals (3) about FH which contributes to underdiagnosis and treatment (2). There are two forms of FH—Heterozygous FH (HeFH) and Homozygous FH (HoFH). HeFH prevalence has been estimated to be around 1 in 300 people (4). Recent reviews have (5, 6) noted the difference in prevalence in global subpopulations and the importance of understanding its true prevalence in the community to target interventions. Previous studies (3, 7, 8) have identified a need to develop a universal screening process to promote early identification and treatment to prevent severe cardiovascular disease and recurrent and pre-mature cardiovascular events (9).

The clinical diagnosis is based on an elevated cholesterol, LDL >4.9 mmol/l, with a triglyceride (TG) concentration within a normal range, and a premature family history of CVD (2, 10). Local guidelines for diagnosis may differ slightly, however the Dutch Lipid Clinic Network Score (DLCNS)or Simon Broome Criteria are commonly accepted criteria to aide in diagnostics (2, 11). Internationally, the former is more frequently used, however national differences in diagnosing and managing FH (1). Genetic testing may be used to diagnose FH, although there are variations in the genetic mutations that result in the disorder (12). Ergo, it is possible to receive a negative genetic test result yet have a phenotypic diagnosis, and vice versa (13).

The Irish Heart Foundation estimates at least 10,000 people in Ireland have FH, and most cases are likely to be undiagnosed (14). The Irish College of General Practitioners (ICGP), the professional body for general practitioners (GPs) in Ireland, estimates this figure could be closer to 20,000 if the estimated Irish prevalence of 1 in 250 is applied (4). Most general practices will have approximately 10–20 undiagnosed cases at conspicuously high risk of early severe vascular disease. With Irish people attending their GP an estimated 4.34 times a year (15), there is an opportunity for primary care clinicians to diagnose and help manage cases of FH in their practices.

Research in Australia, Europe, and the UK (16–19) has suggested that this high-risk group of patients is accessible in the general practice setting and educational resources such as webinars could increase awareness among clinicians. The overall aim of our project was to test the feasibility of a model of care that would include electronic record screening, clinical assessment, and coding of possible FH patients across a network of general practices in Ireland. In addition, a secondary aim was to gauge the awareness and knowledge of FH in the Irish general practice setting.

## Methods

### Recruitment and initial survey

Practices were invited *via* an ICGP member newsletter in January 2021. Before commencing participation, practices were provided with an information leaflet and completed a consent form in accordance with ethical standards. Ethical approval for the study was obtained from the ICGP Research Ethics Committee. After expressing an interest in participating in the project, one survey per practice was requested, completed by either a GP or practice nurse (PN). The survey aimed to assess current awareness and management of FH. Our target was 20 practices based on practical feasibility; while 28 practices expressed an interest, 18 completed the initial survey and hence were included in latter phases.

The survey included questions on practice demographics, educational needs, and quiz style questions (one correct answer). Part of the survey included a questionnaire originally developed by Bell et al. (20) for a 2014 study looking at the knowledge, awareness, and treatment of FH by Australian GPs. It was also used in the FH “Ten Counties Study” (21), and has been used and validated widely including in the UK, India, Saudi Arabia, and Malaysia (22).

For this project, the questions were adjusted for an Irish context, and it was piloted by an Irish GP team. Analysis of survey responses was completed using Microsoft Excel and SPSS V.27, where descriptive statistics were used as well as chi-square tests where appropriate to measure correlation. A *p*-value < 0.05 was considered significant.

### Retrospective electronic health record (chart) review

Practices were asked to search their electronic health record (EHR) database for active patients who had ever had a recorded LDL cholesterol (LDLc) of 4.9 mmol/L or higher. Active patients were defined as either public or private patients who had attended the practice at least once in the past 3 years. Up to thirty patients with the highest LDLc levels were selected for a retrospective chart review, where GPs and/or PNs looked at factors such as family history, smoking status, history of lipid lowering medication, and other health factors.

### Active patient review

After the retrospective chart review, up to ten patients from each practice with the most concerning LDLc levels and who consented to be reviewed underwent an active review. GPs were asked to gain more insight on the patients' history and record any actions taken in relation to FH management. There were sixteen questions in the active review, including questions on co-morbidities, CVD risk factors, information needed for a complete family history, any new diagnostic tests (in relation to FH) and their results and other changes to the patient's care relating to lipid management. Responses were used to assess the possibility of FH diagnosis.

### Educational component

The educational component consisted of a 1 h live virtual webinar with related resources material hosted on the ICGP education platform and available to all participating practices on an ongoing basis. There was no follow up assessment after the session, rather practices were asked to apply the knowledge in the next phases of the study. The educational component occurred after recruitment and initial survey and before the chart review. Our cardiovascular clinical lead also discussed cases with individual practices on request.

## Results

### Survey results

Eighteen practices completed the survey; two of which are single handed practices. In two cases, more than one staff member responded from the practice for a total of 20 responses—for staffing questions, the first completed survey per practice was included; all responses were retained for the awareness questions.

All practices had a minimum of one PN on at least a part time basis, with an average of 2.1 full-time equivalent (FTE) PNs employed across practices. There was a range of 0.6 FTE PNs to 6 FTE PNs per practice. Over 60 individual GPs were employed, for an accumulative 53.5 FTE GPs. Practices were in twelve different counties and were geographically disparate. Half of all practices were in towns. Table 1 covers the demographics of the practices and respondents of the survey.

**Table 1:** Demographic profile of respondents.

	***n***	***%***
**Years in General Practice (*****n** **=*** **20)**		
4–6 years	6	30.0%
7–10 years	1	5.0%
11–15 years	5	25.0%
15+	8	40.0%
**Location (*****n** **=*** **18)**		
A City (50,000+ population)	6	33.3%
A Town (1,500-49,999 population)	9	50.0%
A Village (< 1,499 population)	3	16.7%
**Number of Staff (*****n** **=*** **18)**		***Mean***
Total GPs	68	3.78
Total PNs	40	2.22
FTE GPs	53.5	2.97
FTE PNs	36.9	2.05

A total of 84,936 patients were noted across the eighteen practices, with an average total practice size of 4,718 patients per practice. The maximum number of patients at any one practice was 20,000 and the minimum was 800.

#### Awareness of FH

Respondents were asked to rate their personal level of familiarity with FH on a scale of one to five, where one equals below average and five equals above average, three was average. Across the twenty responses, the average score was 2.65 which is slightly below the “average” level of familiarity. Respondents were asked to rate their practice's overall awareness and knowledge of FH, using the same scale as above. In this case, the practices were considered to have better overall awareness with an average score of 2.75.

When asked if they were aware of any guidelines on the detection and management of FH, around a third of respondents were not aware of any such guidelines. Ninety five percent (*n* = 19) correctly identified that FH is characterized as ‘a genetic disorder with very high cholesterol and a family of premature heart disease. Seventy percent (*n* = 14) of respondents were able to correctly identify the correct lipid profile of someone with FH.

Respondents were presented with a series of options and asked which would assist them in detecting FH in their practice (Table 2). Respondents were able to select as many options as desired. The most frequently selected option was a laboratory report (77.8%, *n* = 14), and a finder tool in their clinical software was a close second (72.2%, *n* = 13).

**Table 2:** Tools to assist in detection of FH.

	**Percent**	**N**
Laboratory report on a lipid profile alerting possible familial hypercholesterolaemia	80.0%	16
Alert by the clinical software system in your practice	60.0%	12
Direct telephone call from the laboratory	30.0%	6
Finder tool in your clinical software system for patient who may meet criteria	70.0%	14
None of the above	0.0%	0
Do not know	5.0%	1

Regarding the prevalence of FH in Ireland, 45.0% (*n* = 9) of respondents correctly identified the estimated prevalence, 25.0% (*n* = 5) said they did not know, and the remaining responses were over or underestimates.

In terms of the likelihood that first degree relatives of someone with FH will also have it themselves, sixty five percent (*n* = 13) of the respondents correctly identified that there is a 50% chance of a patient with a first degree relative with FH having it themselves. The next question asked how much of a greater risk of premature coronary heart disease people with untreated FH have compared to the general population-−45.0% (*n* = 9) correctly identified the risk is 10 times greater, while 25.0% (*n* = 5) did not know and the remaining selected incorrect answers.

Respondents were asked to identify the age for males and females when heart disease is considered “premature”; the correct answer for males was 55 and females 65. Three people (15.0%) did not know for either males or females. A fifth (*n* = 4) correctly identified the age in males. Only 10.0% (*n* = 2) people identified 65 as the threshold in females. Most responses were < 65 years old. Regarding whether an accurate FH diagnosis can only be made after genetic testing, 35.0% (*n* = 7) of the 20 respondents correctly selected “false” as their response while 30.0% did not know.

#### Management of FH

Respondents were given a list of five care options and asked if they routinely carried them out for patients with premature CHD. Overall, 85% (*n* = 17) said they would check the patient's lipid levels, 75.0% (*n* = 15) said they would take a detailed family history (Table 3).

**Table 3:** Routine care activities used for patients with documented premature CAD.

	**%**	***N***
Check patient's lipid levels	85.0%	17
Look for arcus cornealis	25.0%	5
Look for tendon xanthomata	30.0%	6
Take a detailed family history of coronary artery disease	75.0%	15
Screen close relatives for hypercholesterolaemia	25.0%	5
All of the above	25.0%	5
None of the above	0.0%	0

Practices also provided the number of adult patients who had been formally diagnosed with FH, and those who have ever had an LDLc above 4.9 mmol/l. Out of the eighteen practices, two could not search for these factors on their practice software and one practice provided estimates based on prevalence.

The range of total adult patients ranged from 600 to 14,000, with an average of 4,070 adults registered to each practice. For the number of formal diagnoses of FH, the range was from 0 to 50 and the average number per practice was eight. Finally, the number of adults who had ever had an elevated LDLc recorded ranged from 0 to 366 with an average of 133 per practice. Overall, there were a total of 55,205 adult patients at the sixteen practices with valid information, 0.2% of them had a formal FH diagnosis while 3.9% had an LDLc above 4.9 mmol/l at some point.

When asked if there were patients with FH under their care, would the clinician conduct routine screening of close relatives by completing a lipid profile for them. Almost half (45.0%, *n* = 9) of the clinicians did not routinely screen close relatives (Table 4).

**Table 4:** If you have patients with familial hypercholesterolaemia under your care, do you routinely screen close relatives for this condition with a lipid profile?

	**%**	***N***
Yes, patient's children only	0.0%	0
Yes, patient's children and other close relatives	35.0%	7
Refer for Screening	10.0%	2
Screening not available	5.0%	1
No	45.0%	9
Not applicable, I do not have patients diagnosed with FH	5.0%	1

Respondents were asked to identify which healthcare provider would be most effective to detect and diagnose FH, and screen first degree relatives. They were given the option of lipid specialist, GP, cardiologist, specialist nurse, and endocrinologist to choose from for each. Three quarters (*n* = 15) selected GP as the most effective healthcare provider to detect a “significant lipid abnormality,” 45.0% (*n* = 9) selected lipid specialist as the most effective provider to diagnose FH, and 40.0% (*n* = 8) selected specialist nurse as the most effective person to screen family relatives for FH.

In terms of what age should a patient be tested for hypercholesterolaemia in a family that has FH, 40.0% (*n* = 8) said they would start testing at age 13–18 years old, 25.0% (*n* = 4) said they would start testing at age 7–12 and the remainder selected none of the above or do not know. Forty five percent of respondents (*n* = 9) did not know of any specialist services for lipid disorders they could refer to, 45.0% (*n* = 9) were aware of a service and had referred to it, and 5.0% (*n* = 1) said they were aware of a service but have not referred to it and the same number were aware of such a service but did not know if any referrals had been made.

Respondents ranked the key barriers to detection and management of FH in Ireland from 1 to 6. Just under two-thirds of respondents selected ‘Lack of resourced programme in general practice for detection and management' as the most common barrier (Table 5).

**Table 5:** Common barriers to the detection and management of FH.

	**1 = most** **common**	**2**	**3**	**4**	**5**	**6 = least** **common**
Lack of resourced programme in general practice for detection and management	60.0%	5.0%	20.0%	0.0%	5.0%	10.0%
Lack of specialist services	15.0%	25.0%	20.0%	5.0%	10.0%	25.0%
Lack of family screening services	15.8%	15.8%	42.1%	15.8%	10.5%	0.0%
Lack of genetic testing services	20.0%	10.0%	10.0%	25.0%	20.0%	15.0%
Lack of education on this topic for GPs and practice nurses	25.0%	10.0%	35.0%	5.0%	25.0%	0.0%
Lack of guidelines for GPs and Practice Nurses	20.0%	35.0%	25.0%	5.0%	5.0%	10.0%

Each respondent could select multiple options from a list of drugs they would use to treat hypercholesterolaemia in their own practice. All respondents said they would use statins to treat hypercholesterolaemia, over three-quarters indicated they would use ezetimibe (80.0%, *n* = 15), the remainder are shown in Table 6.

**Table 6:** Which drugs would you use to treat hypercholesterolaemia in your practice?

	**%**	***N***
Exchange resins/bile acid sequestrants	5.00%	1
Ezetimibe	80.00%	16
Statins	100.00%	20
Fibrates	35.00%	7
Nicotinic acid	0.00%	0
PCSK9 inhibitors	5.00%	1
None of the above	0.00%	0

#### Learning needs

Practices were asked if they had any learning needs relating to FH. All practices said they had learning needs related to FH. When asked what the best method to fulfill this need, webinar and ICGP guidelines were selected by 80.0% (*n* = 16) and 85.0% (*n* = 17) respectively.

### Educational component

The ICGP Education department collaborated with one of the authors—Joe Gallagher, ICGP HSE Primary Care Lead for Integrated Care Programmes (cardiovascular disease)—and the ICGP Research Department to deliver the educational component.

We recorded individual subject matter experts on the named areas/topics below. All modules were delivered *via* a live webinar followed up by a Question-and-Answer session with Gallagher. A total of 16 practices participated in the live event where both GPs and nurses took part.

These short recordings were then combined as lessons in an overall module, which continue to be available to staff in the participating practices on the ICGP Education platform. This allows for reference to the material at a time that works best for all practice staff. We also provided a discussion facility so that participants can ask questions, share progress etc.

### Retrospective chart review

**Figure 1:**
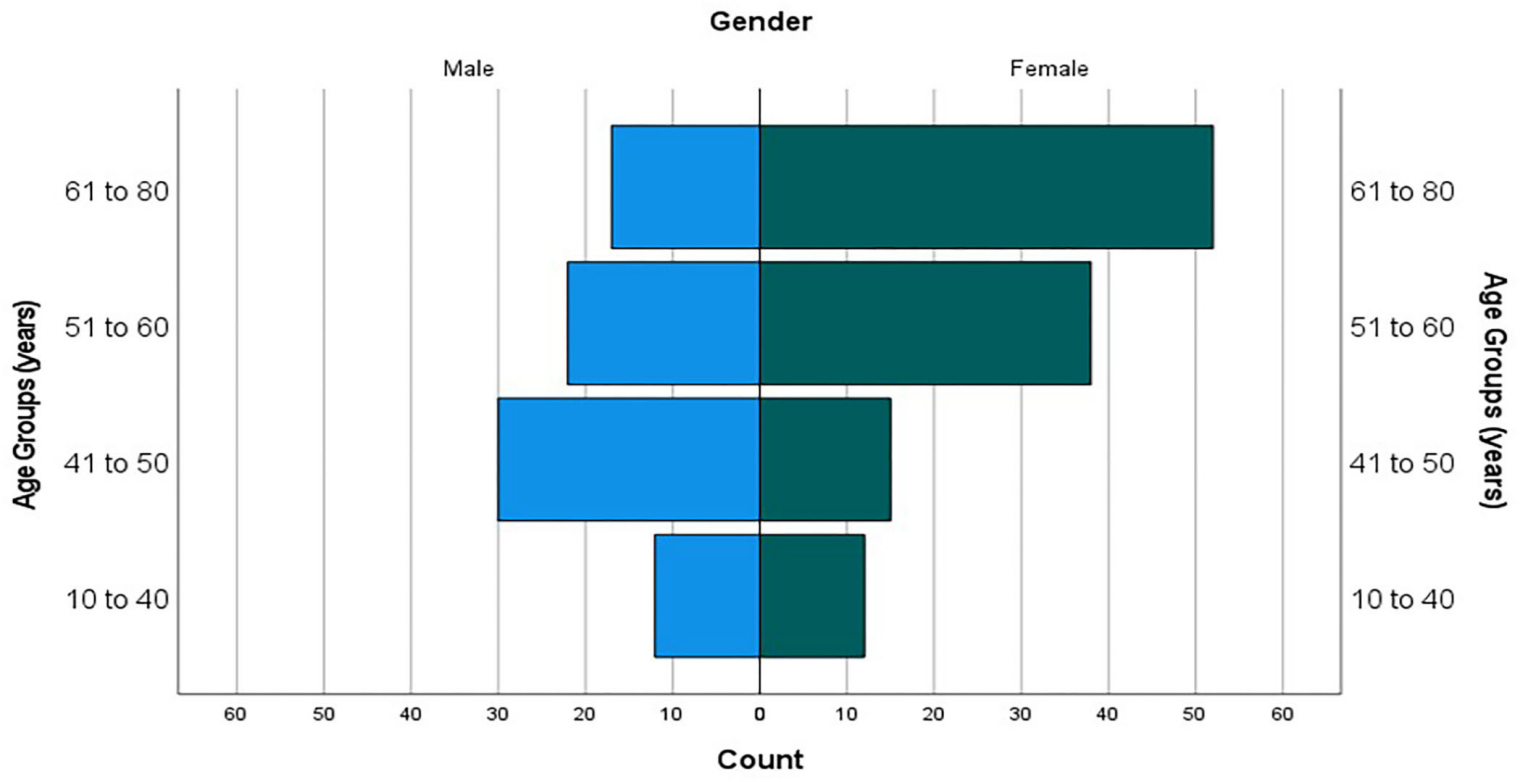
Age pyramid (counts).

Eight practices returned information on 198 patients. The range of the number of patient charts reviewed per practice was 10 to 30. The majority, 59.1%, of patients were female. Just under two-thirds of patients were aged 46–65 years old. The mean age for all patients was 55 years, however female patients tended to be older with a mean age of 57.5 compared to 51.2 for males as there was a higher proportion of males aged 41 to 50 (Figure 1).

Out of the 198 patients, 29.8% had a family history recorded in their electronic medical record—with a higher proportion of male patients (37.0%) having a family history recorded compared to female patients (24.8%). Although not statistically significant, patients who had a family history recorded had higher average LDLc– 4.44 mmol/l compared to 4.39 mmol/l for latest LDLc recorded and 6.00 mmol/l compared to 5.78 mmol/l for highest ever. Just under a quarter of all patients (*n* = 44) had a family history of CVD recorded, and for half of these the family history of *premature* CVD (defined as Female relative < 65 or male relative < 55 years of age) was recorded. Only 7.6% of patients had a personal history of atherosclerosis recorded.

**Figure 2:**
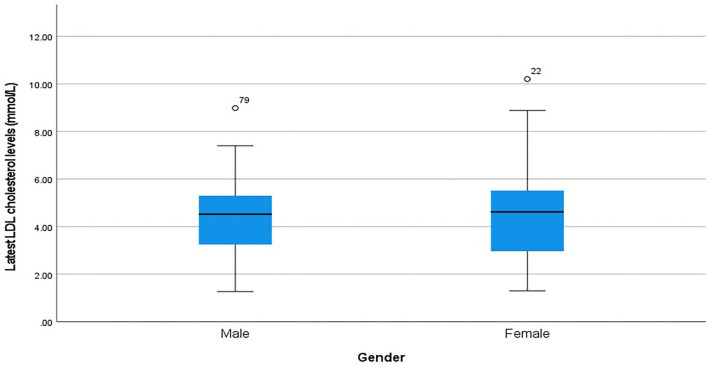
Comparison of the most recent LDLc mean for males and females.

For the highest recorded LDLc, the average was 5.8 mmol/L. Female patients had a slightly higher average of 5.9 mmol/L compared to males at 5.7 mmol/L. In terms of the latest LDLc recorded, the overall mean was 4.41 mmol/l and again the female patients had a slightly higher average of 4.42 mmol/l compared to males at 4.38 mmol/l (Figure 2).

Less than one percent of patients had a Dutch Lipid Clinic Network Score (DLCNS) recorded, while 9.1% of patients reviewed had a diagnosis of FH already.

Female patients also had a higher average BMI of 30.4 compared to males at 29.6 and the overall average of 30.1.

The mean systolic blood pressure (SBP) recorded across all 198 patients was 131.7 mmHg and the mean diastolic blood pressure was 81.3 mmHg. Female patients had an average blood pressure of 132.6/80.9 mmHg and male patients had an average of 130.4/82.1mmHg.

Smoking status was recorded for 68.2% of patients – 38.3% of male patients were current or ex-smokers compared to 23.1% of female patients. Drinking behaviors were less well recorded, with only a third of patients having this information in their records. Out of the patients with drinking frequency recorded, around half said they never drank or drank once a month or less. However, just under one fifth (18.5%) of patients with alcohol consumption information drank four or more times in a week. A third of these patients were drinking more than ten units of alcohol on each occasion.

Regarding the management of FH, GPs reported on medication history and specialist referrals relating to lipid management. Most patients had been or were currently taking statins, while few had experience with ezetimibe or other lipid lowering medication (Table 7). Furthermore, 6.1% of patients had ever been referred to a specialist for lipid management.

**Table 7:** Prescriptions for lipid management.

	**Has the patient ever been prescribed statins?**	**Is the patient currently on statins?**	**Has the patient ever been prescribed ezetimibe?**	**Is the patient currently prescribed ezetimibe?**	**Is the patient on other lipid lowering therapies?**	**Has the patient attended specialist clinic related to their lipids?**
Yes (%)	68.2	58.6	11.1	9.6	1.5	6.1
No (%)	31.3	40.9	88.4	89.9	98.0	93.4
Not recorded (%)	0.5	0.5	0.5	0.5	0.5	0.5

### Active patient review

Seven practices returned data from the patient active review activity—hence seventy patients underwent this targeted review. Within this subset of patients, 64.3% were female and the overall average age was 55.2. The male's mean age was 52, while it was 57 for females. All adult patients' records were checked for family history of CVD, 41.4% of patients had family history information added to their file.

More than one third, 38.6%, of these patients had a comorbid condition. Of these 27 patients, 48.1% (*n* = 13) had hypertension, 22.2% (*n* = 6) had hypothyroidism, and three people had diabetes (Table 8). Nearly half of females (46.7%) in the subset had a comorbidity compared to around one fifth of male patients (21.7%). The mean age for females with comorbid conditions was 58.4 years, for males this was 51 years.

**Table 8:** Patients' comorbid conditions.

	**Percent of Patients**	***N***
No	61.4	43
Yes	38.6%	27
**Frequency of comorbid conditions (% of Yes)**
Hypertension	48.2%	13
Coronary Artery Disease	7.4%	2
Diabetes	11.1%	3
Cardiomyopathy	3.7%	1
Other	37.0%	10

Overall, 60% of patients (*n* = 42) had other CVD risk factors. The most frequently cited risk factor for these patients was smoking, followed by being overweight, having hypertension or a sedentary lifestyle (Table 9). Additionally, we identified a small number of patients with hypothyroidism which may also increase LDL cholesterol, however, the data did not permit us to establish if the highest LDLc recorded was when patient was being treated for hypothyroidism. Two of these patients already had an FH diagnosis.

**Table 9:** Patients' CVD risk factors.

**Risk factor**	**Percent of patients with CVD risk factor with this risk**
Overweight or obesity	38.1%
Hypertension (HTN)	23.8%
Smoker	40.5%
Sedentary	9.5%

Some patients had multiple risk factors and others had some that were unique to them and not included in this table, such as an autoimmune condition. Around half of male patients had at least one CVD risk factor compared to just under two thirds of female patients. As a result, many of the patients were referred for blood tests, had a medication change and/or were given smoking cessation and lifestyle (diet/exercise) advice to help improve their situation. In two cases, a patient's children were notified so they could have their own health check.

New diagnostic tests were ordered for 65% of patients, all of which received new blood tests. In addition, one patient had a 24h BP monitor and another had an ECHO and Carotid Doppler.

In total, thirty-five patients had a new LDLc result; the average overall was 3.83 mmol/l. The lowest result was 1.3 mmol/l and the highest was 8 mmol/l. The average values for each sex were similar, with females having an average of 3.84 mmol/l and males 3.85 mmol/l.

**Figure 3:**
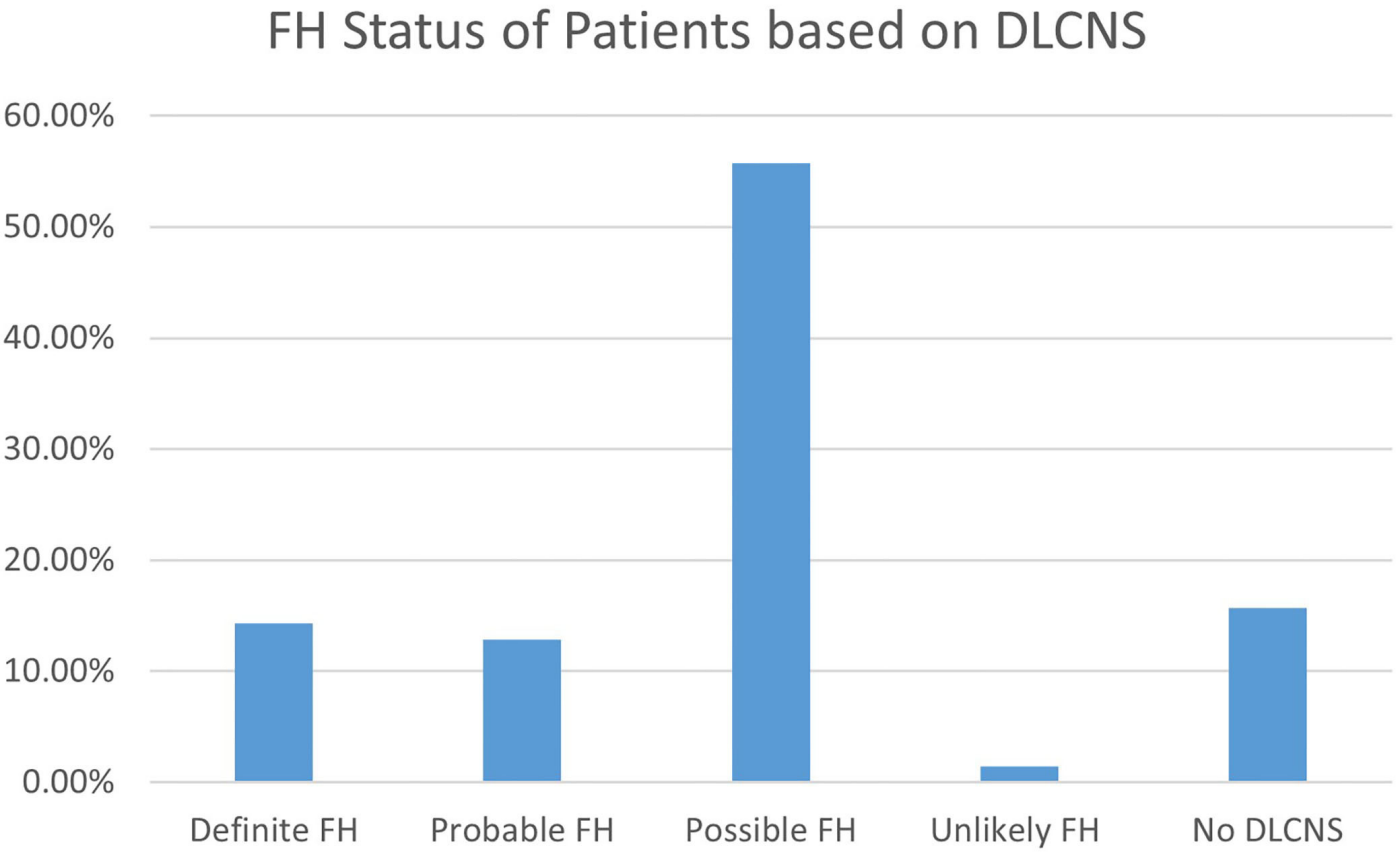
FH status based on DLCNS from active review.

Fifty-nine patients had a DLCNS calculated as part of this review. The female average DLCNS was 4.8 and male was 5.5—overall mean was 5. A DLCNS between 3-5 is considered as “possible FH.” Based on the DLCNS guidelines, 14.3% of these patients were classed as “definite FH” cases (Figure 3).

There were no correlations between age, sex, or whether the patient had other general comorbidities and the newly determined FH status. However, there was a statistically significant (*p* = 0.002) relationship between newly determined FH status and whether the patient had other CVD risk factors.

Some patients had their medications changed (Table 10), most of the medications added were either statins or ezetimibe. Almost a fifth of patients began a new medication because of their review.

**Table 10:** Medication changes.

**Medication changes**	**% of patients**
Decreased	4.29%
Exchanged one medication for another	2.86%
Increased	4.29%
No change	68.57%
Started a new one	17.14%
Stopped	1.43%
N/A	1.43%
Total	100.00%

Finally, around a third of patients were referred to a new specialist service because of the review. The most common referrals were to cardiology or endocrinology. One person was referred to a lipid specialist.

## Discussion

### Summary of results

Out of the 55,205 adult patients at the sixteen practices with valid information, 0.2% of them had a formal FH diagnosis and 3.9% had an LDL above 4.9 mmol/l recorded in their EHR. Very few respondents reported themselves as having an above average familiarity with FH, though 65.0% considered their knowledge to be “average.” Seventy percent of respondents were aware of some guidelines about FH, however, more ICGP guidelines were requested to meet learning needs by 85.0% of the sample. Almost all respondents were able to correctly describe the characteristics of FH and the majority were able to correctly identify a lipid profile that is consistent with a FH diagnosis. The respondents knew which medications to use to treat FH and the importance of taking a family history for these patients, but half had not performed or referred to screening for family members of patients with FH.

Furthermore, as the recording of FH and number of formal diagnoses was low, this may indicate that increased awareness about the importance of early diagnosis and treatment is needed. Gaps in knowledge about the prevalence, risks of untreated FH and the best secondary care services to refer to for help in managing and screening for FH were identified and covered in the educational material—of which sixteen practices participated in a webinar and 13 used the additional online module. This material can now be disseminated to all Irish general practices.

Additionally, 198 adult patients from eight practices with a history of elevated LDLc in Ireland had their charts retrospectively reviewed. Over half (59.1%) of patients were female, and the average age of the patients was 55 years old. Just under two-thirds of patients were aged 46–65 years old. Female patients included were older than males. The key data points from the retrospective chart review were the latest and highest LDLc results. The average latest LDLc result (likely a managed cholesterol score) was 4.4 mmol/l and the highest average LDLc was 5.8 mmol/l. Females tended to have a higher result for LDLc and BMI results, however more males were current smokers.

Seventy patients from seven practices underwent an active health review, just over a quarter of these were subsequently classified as “probable” or “definite FH” using DLCNS criteria. Overall, 41.4% of these patients had their family history updated on their record. All patients were given advice on how to manage their lipids and cardiovascular health, with some starting on medication and others having medication adjusted; all received lifestyle advice to help reduce other identified risk factors. The most frequently identified risk factor for these patients was smoking, either tobacco or vape. Furthermore, just under 40% of these patients had another health concern such as hypertension. Forty-six (65.7%) had new blood tests and 31.4% were referred to a specialist. Half of these patients had a new LDLc result; the average overall was 3.83 mmol/l. The lowest result was 1.3 mmol/l and the highest was 8 mmol/l. This may indicate some patients needing further management of their lipids.

### Strengths and limitations

There were a few challenges and limitations of this work. The initial call for expressions of interest was sent to ICGP members in early 2021, with the goal of recruiting 20 practices. After this email went out, there were 28 interested practices, however due to the time commitment required and the demands of the COVID-19 vaccination schedule that GPs were asked to complete at the same time, along with usual practice duties, there was a gradual drop out over the course of the project. In the end, 18 practices responded to the survey, 16 engaged with the educational materials, 8 completed retrospective chart reviews and finally 7 completed active patient reviews. The small number of practices is considered a limitation for this study; furthermore, it may have introduced a bias as these practices had an interest in the topic of FH, which may have also been true for the patients who participated.

Despite the small number of practices, a strength of this study was that 198 patients had their charts reviewed and 70 had an active investigation into their dyslipidemia. Furthermore, it is a similar sample to a study completed in the UK (23). Another limitation of the study was the limited search capabilities of the practice management software systems in Ireland. Practices had different software packages with variable data quality, some GPs found it difficult or time consuming to search for patients based on recorded LDL cholesterol level.

GPs who discontinued their participation reported being too busy in their practice to participate and this may have introduced bias.

Many of the practices faced challenges in recruiting patients for active reviews, partially due to reluctance to come into the practice during the pandemic and partially due to disinterest in FH from patients. Although others have also found that importance of cholesterol control has not garnered the same attention as blood pressure in the public (17).

Finally, when practices were asked to participate in a final educational activity and qualitative interviews—for both those who had and had not participated throughout the project—there was a lack of interest to continue participation. A possible reason for lower participation than desired could be a low level of awareness and interest in FH among Irish GPs. However, we do not consider that this impacted on the data reported here.

### Comparisons to other literature

In 2014, Bell et al. (20) developed the original questionnaires to determine GPs' knowledge regarding familial hypercholesterolaemia in Western Australia (col 2, Table 11). This questionnaire was then used in other studies, most notably in the “Ten Countries” study (21) (col 4-12, Table 11) led by the FH Australasia Network (26). In that study (21), the UK (25) was used as an international benchmark—these results will also be used. The work has expanded to at least fifteen countries and into other areas of medicine outside of general practice. In the following section, we will compare our key findings from Table 3 to the same table from previous studies. In 2019, Mirzaee et al. (24) repeated the survey with 121 healthcare professionals (HCP) involved with the management of acute coronary syndrome (col 3, Table 11).

**Table 11:** Awareness and knowledge survey response comparisons.

**Awareness**	**1. Ireland**	**2. Western Australia [2014 (20)]**	**3. Cardiac HCPs Victoria, Australia 2019 (24)**	**4. Australia [2017–(21)]**	**5. Japan**	**6. Malaysia**	**7. South Korea**	**8. Philippines**	**9. Hong Kong**	**10. China**	**11. Vietnam**	**12. Taiwan**	**13. UK (25)**
Familiarity of FH rated as above average (>4) (personal)	5%			32%	23%	38%	28%	34%	50%	23%	49%	47%	39%
Familiarity of FH rated as average (3) (personal)	65%	62% (average or above)	76%										
Awareness about FH guidelines	70%	33%	43%	36%	47%	35%	34%	N/A	43%	8%	28%	53%	61%
Awareness about lipid specialists	55%	62%	36%	51%	33%	34%	30%	31%	40%	12%	39%	57%	50%
**Knowledge**													
Correctly described FH	95%	80%	63%	72%	77%	86%	51%	73%	62%	75%	65%	60%	89%
Correctly identified lipid profile	70%	68%	68%	59%	85%	65%	57%	48%	51%	85%	45%	61%	74%
Correctly identified prevalence of FH in the community	45%	27%	16%	26%	41%	24%	19%	16%	11%	17%	14%	30%	30%
Correctly identified the transmission rate of FH to first-degree relatives	65%	45%		44%	40%	49%	42%	37%	49%	36%	26%	61%	51%
Correctly identified the cardiovascular disease risk in untreated FH patients	45%	29%	48%	14%	13%	9%	8%	10%	7%	4%	2%	5%	14%
Correctly identified that genetic testing was not required to accurately diagnose FH	35%	50%		50%	52%	47%	64%	68%	38%	38%	58%	24%	52%
Selected statins to best treat hypercholesterolemia	100%	95%		89%	85%	96%	90%	95%	93%	95%	75%	95%	94%
Selected a combination of statin and ezetimibe to treat severe hypercholesterolemia	65%	74%		64%	48%	56%	70%	48%	49%	77%	31%	63%	50%
**Practice**													
Screened patients with premature CAD for family history	100%	56%	45%	93%	83%	95%	89%	92%	95%	94%	85%	95%	90%
Performed/referred for routine family screening of patients with FH (if GP has FH patients under their care)	45%	53%	8%	86%	30%	82%	50%	53%	90%	47%	83%	77%	73%
The most prevalent age for screening young people in a family with FH was 13–18 years, which was selected by	40%	52%	43%	52%	18%	52%	54%	52%	48%	16%	33%	20%	45%
Have referred FH patients to a lipid specialist (if aware of lipid specialist)	45%	27%		66%	26%	52%	57%	32%	86%	86%	49%	100%	72%
**Opinions on detection**													
Selected GPs as the most effective healthcare provider for the early detection of FH	75%	84%	72%	80%	45%	92%	71%	58%	76%	8%	23%	50%	82%

None of our participants had previously completed the questions ergo it can be considered a baseline awareness level for these practices (Table 11) like the other studies. This table compares our findings to other international results

In our study, 65% of respondents rated their familiarity with FH as average or above with 5% of this being “above average.” In Bell et al.'s 2014 cohort of 191 GPs, 62% rated themselves as average or above (20); in the “Ten Countries” study (21), 34% rated their familiarity above average and 39% were above average in the UK. In comparison with the 2019 Australian results (24), 76% of these HCPs considered their familiarity with FH as average or better. This shows some disparity in above average familiarity with FH in Ireland. Seventy percent of the Irish cohort were aware of guidelines about FH, this was similar to the 61% in the UK (25), and higher than Bell et al.'s Australian GPs at 33% (20), and the 35% in the “Ten Countries Study” (21), and 43% of HCPs inn the Mirzaee et al. 2019 study. In a Croatian study, only 56.9% of the interviewed physicians actively used guidelines in their work, and they found primary care physicians were more likely to rely on their own experience compared to specialists (27). However, ICGP guidelines were the second most requested learning material in our study which could mean that while Irish GPs are aware of guidance on FH, they need more information on the condition. Half of the Irish GPs were aware of lipid specialists, compared to 62% in Bell et al.'s cohort, the same in the UK cohort, and the 35% percent in the Asia-Pacific countries and 36% in Mirzaee's study.

Looking at the knowledge indicators, 95% of our sample were able to correctly identify the FH definition. In comparison, 89% of the UK sample, 63% of the Australian HCPs (24), 80% of the Australian GPs, and 72% of the Asia-Pacific group were able to identify the correct definition. Thirty-five percent of the Irish HCPs correctly identified that genetic testing is not required to diagnose, this was slightly less than the half of GPs in the “Ten Countries” study, Bell et al.'s 2014 Cohort and 52% in the UK study. For treatments, all the Irish clinicals had selected statins as the best option to treat FH, similar to the 94% of the UK GPs had selected this compared to 90% in the “Ten Countries” study and 95% in Bell et al.'s study. However perceived knowledge may differ in practice-−80.6% of physicians in the Croatian study believed they treated patients with dyslipidaemia well, though only 53.3% knew the LDLc target value (27).

Just under half of the Irish respondents performed routine family screening of patients with FH, while similar to Bell et al.'s 53%, this is lower than 73% of the UK respondents, and 66% of respondents in the “Ten Countries” study. More proactive family screening of Irish patients should be conducted. Finally, 75% of the Irish group said GPs were the most effective healthcare provider to detect FH early, the same percent of respondents selects GPs in Mirzaee's study, which is < 84% in Bell et al.'s study and 82% in the UK but more than most of the countries in the “Ten Countries” study.

In a recent study, searching for patients with an elevated LDLc in their electronic health record (EHR) was found to be an effective way to identify the key patients to prioritize when screening for FH (28). These researchers also found that as the LDLc category worsens, using either the DLCNS or Simon Broome technique, so does the presence of secondary causes of dyslipidaemia (28). These are comparable results to what we found, with a significant correlation between a patients' FH status and the presence of secondary CVD risks being observed.

A team of primary care researchers in the UK completed a similar exercise as our study, asking a set of general practices to search EHRs for patients with high total cholesterol (23) and completing an assessment with 118 of these patients. In this study, they also saw females having higher mean cholesterol levels and over a quarter of their patients meeting the Simon-Broome Criteria for possible FH.

Another possible method for identifying patients at risk of FH using EHRs could be employing machine learning techniques. A team of researchers in the United States found that after training their classification tool with information such as DLCNS and total and LDLc measurements, it was able to correctly flag 84% of patients with the highest probability of having FH (29). Other studies (30, 31), have also shown that using and improving clinical tools, such as the Familial Hypercholesterolaemia Case Ascertainment Tool (FAMCAT) (17), in primary care are helpful in finding patients most at risk for having FH ergo increasing diagnoses. This could be an effective method to use in Ireland and elsewhere if the data quality of EHRs is sufficient and contains the required information.

While searching EHRs in primary care has been shown to improve detection of FH (18), it needs to be done at a system level if the thousands of undetected cases are to be identified (17). Ireland currently lacks a formal screening programme (32), and a third of the respondents in this study were unaware of the current available guidelines which may highlight the need for better national awareness of FH. Public awareness should also be considered regarding FH and risks for CVD—in a Croatian survey of the public, 30.9% of people were aware that elevated LDLc increased the risk of CVD (33). The organization “FH Europe” and other key stakeholders aim to implement EU-level policies that will encourage governments to raise awareness and to fund screening programmes and related care (34). This could be an opportunity for Ireland and other EU countries to develop their own screening programme.

Following the detection, standard method of treating FH should also be employed. Santos et al. (12) reported that targeted interventions can reduce the excess mortality resulting from FH, with primary prevention reducing the increased risk of CVD to just two-fold of the general population and secondary reducing the risk to four-fold more than the general population. In our cohort, less than two-thirds of the patients who had ever had an elevated LDLc were currently taking a statin to manage their LDLc—which could indicate an area of improvement in the Irish context.

## Conclusion

The activities in this project have encouraged more in Irish general practice (GPs and PNs) to consider FH when talking to their patients, especially those with an elevated LDLc. Very few patients reviewed had a Dutch Lipid Clinical Network Score or a family history, which would be key elements in improving detection of FH in general practice. Wider awareness in clinicians of how to detect and manage FH for general practice, as we have achieved in this study, can have positive impact on detection and management of FH (18). Using tools such as machine learning algorithms or record flagging may be effective in helping general practice clinicians to identify at-risk patients. Further education and awareness activities for GP staff and possibly a public facing FH campaign could encourage more conversations about it in the doctor's office.

## Data availability statement

The datasets presented in this article are not readily available because all data is anonymous but not available for sharing as per the original Ethics Application. Requests to access the datasets should be directed to claire.collins@icgp.ie.

## Author contributions

CC and JG contributed to the concept and design of this project. JG provided clinical expertise throughout the project, including during the interpretation, and writing of results. RH prepared study materials, worked with participants throughout the project, managed data collection, and conducted analysis with guidance from CC and JG. RH prepared the first draft of the manuscript. All authors contributed to the revision of the manuscript prior to submission.

## Funding

Amgen provided the funding for this project, however, neither Amgen or its employees had any role in the educational content, practice recruitment, data collection or interpretation of data.

## Conflict of interest

The authors declare that the research was conducted in the absence of any commercial or financial relationships that could be construed as a potential conflict of interest.

## Publisher's note

All claims expressed in this article are solely those of the authors and do not necessarily represent those of their affiliated organizations, or those of the publisher, the editors and the reviewers. Any product that may be evaluated in this article, or claim that may be made by its manufacturer, is not guaranteed or endorsed by the publisher.
